# A Pattern Analysis of Gene Conversion Literature

**DOI:** 10.1155/2009/761512

**Published:** 2010-01-31

**Authors:** Mark J. Lawson, Jian Jiao, Weiguo Fan, Liqing Zhang

**Affiliations:** Department of Computer Science, Virginia Tech, Blacksburg, VA 24061, USA

## Abstract

Gene conversion is an important biological process that involves the transfer of genetic (sequence) information from one gene to another. This can have a variety of effects on an organism, both short-term and long-term and both positive and detrimental. In an effort to better understand this process, we searched through over 3,000 abstracts that contain research on gene conversions, tagging the important data and performing an analysis on what we extract. Through this we established trends that give a better insight into gene conversion research and genetic research in general. Our results show the importance of the process and the importance of continuing gene conversion research.

## 1. Introduction

At its most basic level, gene conversion is the exchange of information between two genes [1]. One gene is the donor and gives part or all of its information to another gene, the acceptor. The process is done through strand breaks and sequence exchanges on the biological level and typically occurs between highly similar genes, for instance, genes that are in the same gene family. Naturally, this leads to an alteration in the acceptor gene (the donor remains unchanged). What kind of alteration this leads to depends on a variety of factors. If the acceptor receives all of the donor sequence (and retains none of its own), then it will take on similar functionality as the donor. This is important for maintaining gene conservation as genes can switch being donor and acceptor, undoing mutations that may have altered the sequence in a detrimental fashion (an example can be seen in mammalian *TLR1* and *TLR6* [2]). However, due to the fact that the donor takes on the functionality of the acceptor, problems can occur if the donor is not functional [3]. Then gene conversion can lead to serious diseases and disorders such as congenital adrenal hyperplasia [4] and spinal muscular atrophy [5].

Interesting also is when only a part of the acceptor sequence is replaced. This typically leads to a change in functionality in the acceptor gene and is an important method to achieve genetic diversity. We see this prominently in the human major histocompatiblity complex genes (the *HLA* genes). Here new alleles are created through gene conversions of existing alleles [6]. Gene conversion is also often used to achieve diversity in immunoglobulin genes (as observed in mostly rabbits and chickens) [7]. In these instances a pseudogene is used to donate part of its sequence to the functional immunoglobulin genes, achieving diversity and allowing for adaptation to deal with foreign objects in the organism. This method is also often referred to as “templated mutation” as it alters the gene according to the template of the donor gene.

As demonstrated by our description and examples, gene conversion is an important process for all organisms. Further understanding of gene conversions will lead to advances such as a better understanding of evolution and a better understanding of (and hopefully prevention of) genetic diseases and disorders. A large amount of research has been devoted to gene conversions, which has given much insight into the process and its effects, both short-term and long-term. However, identification of gene conversions is a difficult process as it typically requires comprehensive phylogenetic analyses across multiple species. While programs do exist that are meant to identify gene conversions (GENECONV [8] and Partimatrix [9] are two typical examples), our own research has shown their accuracy to be less than satisfactory [10].

In order to help further understand gene conversions, we have searched PubMed for research on this topic and extracted and analyzed what was contained in the abstracts. Through this we have identified important trends in gene conversions that can help with continued research. Furthermore, we have listed these papers online to allow the reader to access this research with ease.

## 2. Material and Methods

We downloaded abstracts from PubMed [11] which had the term “gene conversion” in them. The downloaded abstracts ranged in years from 1969 through March of 2008 (when the abstracts were downloaded). Each abstract was then stored in its own file. We then went through all the abstracts and manually tagged the following information.

**(i) Biological Mechanism or Event**

At their most basic level, the majority of papers can be broken down into these two categories. If the research is focused on identifying the biological mechanisms of a gene conversion (such as which proteins are involved in the process or under what conditions an organism shows an increase/decrease in gene conversion), then it is labeled as a “mechanism” paper. If the research is more focused on identifying a gene conversion event (such as an ancient gene conversion between two genes or two alleles), then it is labeled as an “event.”

**(ii) Interallelic or Intergenic**

A key characteristic in gene conversions is between what two genes the gene conversion occurs, that is, did the gene conversion occur between two distinct loci (“intergenic”) or did it occur between two alleles of the same gene (“interallelic”)?

**(iii) Genetic Diversity or Gene Conservation**

While gene conversions can often be detrimental (as we will illustrate when we look at the listing of genetic diseases and disorders associated with gene conversions), there are evolutionary reasons for gene conversions existing. On the one hand, gene conversions can lead to genetic diversity by creating new alleles through combining pieces of other alleles. On the other hand, gene conversion can also lead to gene conservation by having two genes maintain similarity through sequence exchange.

**(iv) Gene-to-Gene or Gene-to-Pseudogene**

In cases where gene conversion occurs between two distinct genes, it is of interest of what type these genes are. Are they both functional, protein-coding genes (“gene-to-gene”) or is one of them a nonfunctional pseudogene (“gene-to-pseudogene”)? Gene conversions involving pseudogenes can have a variety of effects, both good and bad.

**(v) 1-to-1, 1-to-Many, and Many-to-Many**

Essentially, all gene conversions are between two genes. But if we model the evolution of a group of genes over a longer time period, interesting trends can be established within this group in terms of gene conversion. Sometimes, it is still just isolated to two genes (“1-to-1”). Other times multiple genes are involved in gene conversion events. These can be broken down into situations where one gene is used as a donor to multiple other genes (“1-to-Many”) and when gene conversions have occurred amongst a group of genes with no one donor being determined (“Many-to-Many”).

**(vi) Regions and Genes**

Important for this analysis and for future research is a listing of which genes were involved in the gene conversion. When this information is available (this data is not always within the abstract), it is extracted in whatever detail is provided. Sometimes this gives exact gene names (*RAD51*, *RAD52*), other times only a more generic description (“immunoglobulin genes”). There are also occasions where conversions occur across an entire chromosomal region, that can encompass many or even no genes. These were extracted as “regions.”

**(vii) Genic Locations**

Gene conversion does not always encompass the entire gene. In many cases, only a specific region within the gene (for instance the second exon) is involved in the conversion. While this data does not often appear in an abstract, we extracted it when it was available. Specifically we looked for gene conversions that occur within exons, introns, 5′-UTR, or 3′-UTR.

**(viii) Algorithms and Models**

Through our search for abstracts regarding gene conversions, we also encountered those that present means of identifying gene conversions (“algorithms”) and those that discuss how and/or why gene conversions occur (“models”) with this being more of the research's emphasis than the identification of gene conversions. This information was extracted as well.

**(ix) Disease/Disorder Caused**

An important part of gene conversion research is identifying the genetic diseases and disorders that a gene conversion can cause. Due to diseases having different names or abbreviations, we created a unique tag for each disease. So “Congenital Adrenal Hyperplasia” and “CAH” are identified as the same disease. In total 58 unique tags were created, giving us 58 different diseases.

**(x) Species**

The final important data element to extract is in which species this gene conversion occurs. Much like with the diseases, a unique tag was created for each species. So “*Homo sapien*” and “human” are identified as the same species. In total 308 unique tags were created.

All abstracts were read through and tagged using the traditional tagging style used in standards such as XML and HTML. So there was an opening tag (<TAG>) and a closing tag (</TAG>). This way we could extract elements of the text that contributed to this tag being used. The focus was on locating the minimum amount of information that led to the use of this tag. Text such as gene names can be extracted and future research can be done to predict these tags based on this data.

Taxonomy for all species was determined through the taxonomy database of NCBI [11]. Descriptions and pathogenicity of microorganisms was extracted from the Microbewiki [12].

## 3. Results

In total 3575 abstracts were downloaded from PubMed that contained the term “gene conversion.” However, only 2478 were tagged as having information on gene conversions. This is due to a variety of reasons. Some files contained only titles with no abstracts, due to some papers not possessing abstracts or these abstracts not being present in PubMed. Other papers may have dealt with another “conversion” process that also happened to involve genes. This is because the search algorithm uses BOOLEAN “and,” therefore, as long as the abstracts contain both “gene” and “conversion,” the abstracts will be included. And finally, some papers mentioned gene conversions but this was not the focus of the research being presented. For instance, the *AID* gene is important for gene conversion [13] (which in turn is usually mentioned when introducing the gene) but has had other research conducted with it that does not involve gene conversions. In cases where the research does not involve gene conversions, the file was not tagged and excluded from the analyses.

### 3.1. Gene Conversion Species and Taxonomy

In total, we identified 308 unique species. The actual number of species is likely to be higher. Because exact species names are not always given in the abstract, for the cases where no exact species names are given, we counted the species as one as long as the common species names are the same. For instance, many abstracts say they used mice in their research; so the assumption we are going with is that they used the species *Mus musculus*. In these instances, we went with the most commonly used species (like those listed in Ensembl [14] or the UCSC Genome site [15]). Table 1 lists the 25 species with the highest counts in terms of how many abstracts that they appeared in. The order of the species names is not so surprising. Much research is done on humans in general; so they unsurprisingly take the top spot. Furthermore, many of the species listed here are “model organisms” that have had extensive genetic analysis done on them. Virtually all of them have most if not all of their genomes sequenced. In contrast to the species that we have listed here, 199 species (approximately 2/3 of all species) appear in only one abstract.

In addition to the general count, we have listed the count of in what capacity the gene conversion research was done. Was a *mechanism* of gene conversion being studied or was there an identification of a gene conversion *event*? The N/A category is used for situations where it was unclear whether it was either one (for instance situations where the species is mentioned in connection with a model for gene conversion or used as input for a gene conversion identification algorithm). Table 1 shows that yeast, E. coli, and Chinese Hamster were more used for identifying gene conversion mechanisms whereas human, rats, chimps, and cows were more used for identifying events. Some seem to have a balance between the two such as mice, *Drosophila melanogaster*, and rabbits.

Breaking down the species into superkingdoms we found that gene conversion research has been mostly done in eukaryotes (Table 2). In total 247 species are eukaryotes, 40 species are bacteria, and one species is an archaea (*Thermococcus*). In addition, 15 of the species are viruses. Using this species breakdown, we can then see how many papers fall into these categories as well. In total, 2030 papers fall into the eukaryote category, 137 are bacteria, 30 are viruses, and 1 is archaea.

Since eukaryotes are overwhelmingly favored, we further brokedown this superkingdom into three kingdoms. 24 species fall into the fungi category, 154 into the metazoa category, and 44 fall into the viridiplantae category. 589 papers are in fungi, 1268 in metazoa, and 93 in viridiplantae.

Table 2 shows that whereas eukaryotes have a relative equal amount of mechanism versus event research, both bacteria and virus research are more focused on the mechanistic aspects of gene conversion. If we further look at the breakdown of eukaryotes, we can see that there is a difference in the kingdoms in terms of this ratio as well. Both metazoa and viridiplanate researchs tend more towards the identification of events and fungi research tends more towards mechanisms.

We further brokedown the species into classes and orders. In total the 308 species encompass 44 different classes and 94 different orders. The top five classes include Mammalia, Saccharomycetes, Insecta, Aves, and Gammaproteobacteria. Mammalia and Insecta have more event abstracts (704 versus 251 and 91 versus 59, resp.) while Saccharomycetes and Gammaproteobacteria have more mechanism abstracts (476 versus 29 and 48 versus 16, resp.). Aves had a similar amount for both (55 mechanism versus 46 event).

The top five orders include Primates, Saccharomycetales, Rodentia, Diptera, and Galliformes. Primates have a clear bias towards event abstracts (543 versus 117). Saccharomycetales and Galliformes have more mechanism abstracts (476 versus 29 and 54 versus 38, resp.). Both Rodentia and Diptera seem to have a balance between mechanism and event abstracts (110 mechanism versus 125 event and 55 mechanism versus 65 event).

### 3.2. Chronological Trend Analysis

**Figure 1:**
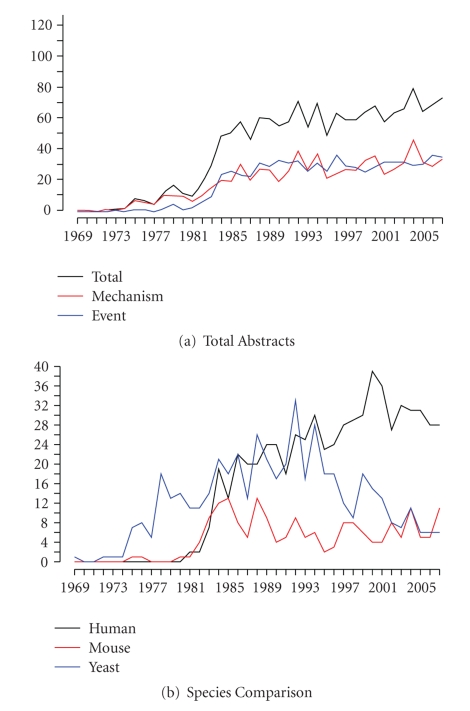
Trend Analysis Graphs. These graphs show the chronological trend of the tagged abstracts. For all graphs, the *x*-axis represents years and the *y*-axis represents the amount of tagged abstracts published in that year. Graph (a) represents the total abstracts published in each year and the breakdown into mechanism abstracts and event abstracts. Graph (b) shows a comparison between the three most studied species: human, yeast, and mouse.

Figure 1 shows the amount of tagged abstracts published in each year (the year 2008 was not included due to not having data for the entire year). As can be seen in Figure 1(a), the overall amount of gene conversion research is growing with each passing year. While there may be drop-offs in some years, the overall trend is towards increased research on this topic. This increase is both in those papers focused on identifying gene conversion mechanisms and those focused on identifying actual gene conversion events. Some years have more mechanism research, some more event research, but an overall increasing trend is quite evident.

To further analyze the chronological trends, we looked at the top three studied species: human (*Homo sapiens*), yeast (*Saccharomyces cerevisiae*), and mouse (*Mus musculus*). While human research exhibits a definite increase, the other species do not. Yeast research on gene conversions seems to have peaked in 1992 and has been decreasing ever since. Meanwhile, mouse research seems to stay at a somewhat consistent level, increasing and decreasing in bursts.

An observable trend that reveals itself in every graph is a large increase in the amount of gene conversion research in the early 1980s. Between the years 1981 and 1986 there is a sharp spike in the total number of gene conversion abstracts. This spike seems to include both mechanism and event research as well as researches in the three species.

### 3.3. Conservation and Diversity

In an almost contradictory fashion, gene conversions are important for the creation of genetic diversity and the conservation of genetic sequences. Table 3 shows a breakdown of these two gene conversion outcomes within the 25 species with the highest abstract count as listed in Table 1. In the table, we compare genetic diversity outcomes with gene conservation outcomes as well as interallelic gene conversions with intergenic gene conversions. A general “rule of thumb” is that interallelic gene conversions lead to genetic diversity (by creating new polymorphisms and thereby new alleles) and that intergenic gene conversions lead to gene conservation (by maintaining homogeneity between two or more genes, undoing mutations). This is evident within our own results. While exceptions do exist, in most cases of interallelic gene conversion, genetic diversity is the outcome and in most cases of intergenic gene conversion, gene conservation is the outcome.

### 3.4. Genes

In total, 1412 abstracts had genes listed that were involved in gene conversion. In addition, 138 abstracts had regions that underwent gene conversion. In this section, we detail the genes that have been extensively studied for gene conversion.

#### 3.4.1. RAD Genes

The RAD family of genes (among them *RAD51*, *RAD52*, *RAD54*, *RAD57*, and *RAD59*) are instrumental in the gene conversion process [16]. They produce proteins that facilitate double-strand repair during meiosis, effectively inserting the donated sequence into the acceptor locus. Because of their importance to gene conversion, they have much research devoted to them. The RAD genes and their functionality are mostly researched in yeast (*Saccharomyces cerevisiae*) and as many as 45 abstracts were devoted to their study in this organism. In addition, research on RAD genes were found in Fission Yeast (*Schizosaccharomyces pombe*) in three abstracts.

Additional species that had RAD genes research done on them are mouse, *Drosophila melanogaster*, chinese hamster, chicken, and rabbit. All of these abstracts focused on mechanistic aspects of these genes.

#### 3.4.2. CYP Genes

The group CYP genes (or genes that are responsible for creating cytochrome P450) contains the gene *CYP21*, a gene whose mutation leads to hydroxylase deficiency which in turn leads to congenital adrenal hyperplasia [4]. As we will show in the disease and disorders section, this is a well-studied gene. Another set of genes (*CYPD6* and *CYPD7*) are involved in a gene conversion that causes debrisoquine polymorphism [17] and a gene conversion between *CYP11B2* and *CYP11B1* has been linked to causing hypertension [18].

In humans it is typically linked to diseases, with over 80 abstracts containing study of this gene in *Homo sapiens*. However these genes have been shown to be involved in gene conversions in other species as well, including mouse, chicken, rat, rabbit, pig, *Helicoverpa armigera*, *Helicoverpus zea*, common cormorant, and collared peccary.

#### 3.4.3. MHC Genes

The major histocompatibility complex genes (MHC genes) play important roles in the immune system [19] and have been linked to sexual selection and compatibility [20]. A commonly studied subgroup of the MHC genes is the human leukocyte antigen genes (*HLA* genes) [6]. These genes are known to have a high level of diversity which is often achieved through interallelic gene conversions. In humans we tagged 136 papers that dealt with *HLA* diversity through gene conversions. In addition, gene conversions involving MHC genes were found in 21 other species, for example, mouse, cow, chicken, goat, sheep, rhesus monkey, owl monkey, lemur, and mandrill.

#### 3.4.4. Immunoglobulin Genes

The immune system must be able to defend an organism from a diverse amount of pathogens. Therefore a large amount of diversity is needed in the genes involved in the immune system and gene conversion is an important mechanism through which high diversity is achieved [7]. This phenomenon is mostly studied in chickens where diversity is created in the bursa of Fabricius [21]. Chickens possess a single functional immunoglobulin light chain *Ig* gene which then undergoes gene conversion with one of 25 pseudogenes to create a diverse amount of B cells. This phenomenon is known as “templated mutation” as the *Ig* gene is being changed (mutated) according to a given sequence. Not surprisingly, chicken was the most common species associated with immunoglobulin gene conversion with 22 abstracts dealing with research on this. Another species that had a large count of *Ig* gene conversion research was rabbit (16 abstracts). The rabbit appendix has been shown to have a similar function to the bursa of Fabricius [22] and achieves *Ig* gene diversity through a similar gene conversion process [23]. Additional species that have gene conversion linked to immunoglobulin genes include humans (17 abstracts), mice (13 abstracts), pigs (3 abstracts), and sheep (2 abstracts).

### 3.5. Diseases and Disorders

In Tables 4 and 5, we can see all genetic diseases and disorders that were linked to gene conversions. In addition to the name of the disease/disorder, we also attempted to extract which genes were involved with the gene conversion and whether one of them was a known pseudogene. In many cases this was difficult to discern. One reason was the shortcoming of extracting this information from abstracts which may not contain exact gene names. Another springs from the fact that the second gene (the donor) may be unknown.

It has been shown that a genetic disease or disorder can involve a gene conversion with a pseudogene [3]. Pseudogenes are genes that are no longer functional due to mutations.

So if part (or all) of a pseudogene's sequence information is transferred to the functional gene, the functional gene may lose function, which might be detrimental to the organism. As can be seen in the disease tables, this is a common cause of genetic diseases/disorders.

However, it is also possible that a gene conversion between two functional genes can lead to a genetic disease/disorder. A slight change in DNA sequence is sometimes all a gene needs to alter its functionality and while the donor gene is generally highly similar to the acceptor gene, there is enough of a difference to be detrimental to the entire organism.

The most studied genetic disease associated with gene conversions is congenital adrenal hyperplasia (OMIM ID: 201910). In our abstract literature analysis, we encountered this disease in 77 papers, although in some papers it was also listed as 21-hydroxylase deficiency. Congenital adrenal hyperplasia can take on many forms but is most often associated with altered production of sex steroids and altered development of sex organs. While it can have many genetic causes, it has been shown to be caused in many situations by a gene conversion between the *CYP21* gene (acceptor) and a pseudogene copy of this gene called *CYP21P* (donor).

### 3.6. Adaptability of Bacteria and Other Microorganisms

Much research has been devoted to gene conversions and bacteria [24]. In total we identified 40 bacteria and as indicated in Table 2, more research has been devoted to studying the mechanisms of gene conversion in bacteria than identifying gene conversion events. Many of these mechanism papers focus on merely identifying what genes and processes have an active role in gene conversion. However, for some bacteria, gene conversion has been shown to actively contribute to adaptation. These abstracts were categorized as having both a mechanism tag and a genetic diversity tag, showing the contents of the abstract dealt with mechanisms to achieve diversity and thus adaptability.

The following bacteria were identified as using gene conversions to achieve adaptation: *Escherichia coli*, *Neisseria gonorrhoeae*, *Streptococcus pneumoniae*, *Borrelia burgdorferi*, *Anaplasma marginale*, *Synechocystis*, *Anaplasma phagocytophilum*, *Neisseria meningitidis pilus*, *Treponema pallidum*, *Borrelia hermsii*, *Mycoplasma genitalium*, *Mycoplasma synoviae*, *Proteus mirabilis*, and *Babesia bovis*. With the exception of *Synechocystis*, all of these bacteria are mammalian symbionts and can be pathogenic in immunodepressed individuals. Three of them are causes of sexually transmitted diseases (STDs): *Neisseria gonorrhoeae* causes gonorrhea, *Treponema pallidum* causes syphilis, and *Mycoplasma genitalium* causes nongonococcal urethritis in men and bacterial vaginosis in women. *Anaplasma phagocytophilum* and *Anaplasma marginale* are responsible for anaplasmosis in humans and cows, respectively. *Babesia bovis* causes Babesiosis (Texas cattle fever) in cows. *Borrelia burgdorferi* and *Borrelia hermsii* are both spirochetes that cause Lyme disease and and relapsing fever, respectively. *Streptococcus pneumoniae* causes pneumonia, *Neisseria meningitidis pilus* causes meningitis, and *Escherichia coli* (commonly referred to as *E. coli*) can cause food poisoning (gastroenteritis). *Mycoplasma synoviae* can cause respiratory tract disease and synovitis in chickens and turkey and *Proteus mirabilis* is a common cause of urinary tract infections. The lone exception is *Synechocystis*. *Synechocystis* is a cyanobacteria and is highly studied [25] due to the fact that it can adapt to its current environment and grow itself photolithotrophically (through photosynthesis) and heterotrophically (through glycolysis and oxidative phosphorylation). It is likely that gene conversion mechanisms figure into this high level of adaptibility [26].

Eukaryotic microorganisms have also been shown to use gene conversion for adaptation, for example, *Trypanosoma brucei*, *Trypanosoma cruzi*, *Toxoplasma gondii*, *Pneumocystis carinii*, *Eimeria tenella*, to name a few. All of these organisms are pathogenic. *Trypanosoma brucei*, also known as the African trypanosome, is a parasitic eukaryote that is spread through the tsetse fly. It causes African trypanosomiasis (sleeping sickness) and is a highly studied organism [27] (it is linked to 34 abstracts). Closely related is *Trypanosoma cruzi* which causes Chagas disease. *Toxoplasma gondii* causes toxoplamsmosis and *Pneumocystis carinii* is a fungus that causes pneumonia. *Eimeria tenella* causes hemorrhagic cecal coccidiosis in young poultry.

### 3.7. Further Analyses

In Table 6 we show further analyses that were determined based on the tagged information. In terms of identified regions that underwent gene conversions, few papers list this within their abstract. This requires an in-depth analysis of the sequence and quite often this is not even listed within the results themselves. Nevertheless, we can see that exons appear to have the highest amount of gene conversions associated with them and that no abstracts were found that had gene conversions in the 3′-UTR region.

As expected, the majority of gene conversions found (in which we were able to clearly establish the amounts) were 1-to-1. This is unsurprising as gene conversion is by definition a process that involves only two genes. However we did find papers that established larger trends of gene conversion, most often involving gene conversion events that lead to conservation. In few occasions (3) one gene is used as the primary donor to two or more genes. More often a group of genes (in 36 abstracts) maintains sequence similarity by engaging in multiple gene conversions over a long period of time.

## 4. Discussion

In total, we found 2478 abstracts in PubMed that detail research on the causes and outcomes of gene conversions. As can be seen by looking at the chronological trends in Figure 1, we can expect the amount of gene conversion research to continue to increase based on the increase of research between 1969 and 2007. And it should increase as well. Gene conversion is an important evolutionary process that is universal across all kingdoms of life and has both beneficial and detrimental effects for an organism. But the research we have is too biased to make clear, absolute inferences on the nature of gene conversions and to predict future gene conversions.

### 4.1. A Universal Process

Gene conversion research can be found in all three superkingdoms (Eukaryota, Bacteria, and Archaea) as well as viruses. The fact that it encompasses all types of life shows how universal the process is. Unfortunately, few research exists on Archaea. Only one abstract is on gene conversion in *Thermococcus*.

The fact that 308 different species were found is also significant. Despite the fact that close to 2/3 of the species appear in only one abstract, we can safely conclude that gene conversion is indeed a wide-spread process. The type of research is also relatively wide-spread as well, 127 species have had gene conversion mechanism research done with them, and 234 species have had gene conversion events identified.

Interesting also is how many species have gene conversions associated with the same genes. 7 species were shown to use RAD genes as part of the gene conversion process (although it is very likely that this number is higher). The CYP genes undergo deleterious gene conversions in humans and 9 other species (although these do not cause any known diseases). Meanwhile, the MHC genes are associated with gene conversions in 22 species and immunoglobulin genes have 19 species that exhibit gene conversions with them. It is therefore very likely that more of these patterns of overlap will be found as gene names become more standardized and listings of orthology increase.

### 4.2. Long-Term and Short-Term Effects

The effect of gene conversion on the evolution of genes can be both long-term and short-term. There are two main categories for the long-term evolutionary effects of gene conversion: genetic diversity and gene conservation. As can be seen by the data in Table 3, our hypothesis holds: an interallelic gene conversion leads to genetic diversity and an intergenic gene conversion leads to gene conservation. In this way, potentially detrimental mutations are undone by replacing a sequence from a highly similar gene (gene conservation) and SNPs of different alleles are combined to create new alleles (genetic diversity).

What is interesting is the exceptions to this rule and more research in this regard could have interesting results. Table 3 shows that more species had an intergenic event that leads to diversity than vice versa. Most likely these conversions only replace part of the gene sequence thus creating a new sequence that combines both acceptor and donor genes.

In total, more intergenic gene conversions (418, encompassing 150 species) were identified than interallelic (208, encompassing 106 species). This is most likely due to the fact that the identification of intergenic gene conversions is relatively easier than that of interallelic gene conversions. Due to the high similarity between alleles, it is often difficult to determine which two alleles combined to make a new one.

The immediate short-term effect that is evident when looking at our results is the amount of diseases and disorders associated with gene conversion research. 58 diseases is a large number and is in fact a larger number than what was found in a recent review of human gene conversions [1]. This discrepancy exists due to the extremely thorough process we used to find gene conversion in the abstract literature and we also included diseases where the exact gene conversion was not found (the Chen review paper lists both genes involved in the gene conversion). Regardless, gene conversions have been shown to cause diseases in humans and future research into their detection and possible prevention is of great importance.

Another short-term effect is the use of gene conversions to achieve genetic diversity. Here we have two competing sides: bacteria and other pathogenic microorganisms that use it to adapt and immune system genes that use it to create diverse B cells to combat those pathogens. While mutation would work as well (and immunoglobulin genes do use hypermutation), this type of “templated mutation” ensures a more structured alteration of the genetic sequence. It is not random like regular mutation, thus ensuring better, quicker results.

### 4.3. Bias of Scientific Research

Out of the 2478 abstracts containing gene conversion research, more than 25% deal with gene conversions in humans. If we count yeast (*Saccharomyces cerevisiae*), then we have over 45% of all the abstracts. And if we factor in the remaining top five organisms (mouse, chicken, and fruitfly), we have over 60% of the total amount of abstracts. Clearly there is a bias in terms of which organisms have gene conversions associated with them. This bias is most likely also prevalent in not only gene conversion research but also in genetic research as a whole.

Humans (*Homo sapiens*) are the most common species in the gene conversion research. This is most likely universal across genetic research as a whole. While some species might be easier to experiment with, human research is directly applicable to ourselves. For instance, in our results we document a listing of diseases and disorders that are caused by/associated with gene conversions. Even research on other organisms can be seen to result from their interactions with us. All of the species listed among those that use gene conversion for adaptation (except one) are pathogenic to humans.

The other species listed here are model organisms. Of particular interest are the yeast species. Three types of yeast are listed in Table 1 (*Saccharomyces cerevisiae*, *Schizosccharomyces pombe*, and *Candida albicans*). As can be seen, they all have more mechanistic research devoted to them than identified gene conversion events (496 abstracts, 41.4% of all the mechanism papers). This is because yeast is commonly used as a model organism for many genetic processes and gene conversion is no exception. In fact, yeast was the first species in which a gene conversion was discovered [28]. While much research is devoted to identifying those genes (such as the *RAD* genes) involved in gene conversions under “normal conditions,” there is a large amount of research devoted to “induced” gene conversions in yeast. When faced with a certain type of substance, increased gene conversions can be seen in yeast offspring. This is due to observable phenotypic changes in the yeast, most often involving the mating type gene *MAT* [29]. Since this increase in gene conversions is detrimental to the organism, the substance is then viewed as genotoxic. Inducing gene conversions in yeast is a commonly used genotoxicity test for a substance (such as the effects of cigarette smoke [30]).

Naturally, there are problems that stem from this bias. If one just looks at the raw counts in Table 1, it would seem that these species have “more” gene conversions than other species. However, just because more research exists on them does not mean that this phenomenon is more widespread in these species than in species with a lower number of abstracts (or even no abstract).

Another problem stems from the detrimental effects of gene conversions. Due to the sheer number of examples, it is clear that gene conversions can lead to diseases and disorders in humans. However, we found no diseases/disorders in other species. Does this mean that gene conversion does not cause these detrimental effects in other species? Also, with one exception, those bacteria and microorganisms that have their adaptation linked with gene conversions can be pathogenic. Is this process more prevalent in pathogenic organisms or is it just that pathogenic organisms are more studied due to the fact that they cause diseases? Clearly more gene conversion research is needed to address these issues and give us a better idea of the whole process. 

### 4.4. Future Work

While we were able to extract a significant amount of data from the abstracts we collected, there were some shortcomings to this process. Oftentimes we were not able to identify the exact species (i.e., “Primates” or “Plants” were listed) and other times exact gene names were difficult to find (if they were there at all). And as can be seen in Table 6 we were unable to extract more details such as the genic region involved in the gene conversion. In addition, we may have missed the papers that focus on gene conversion but have only the equivalent terms to “gene conversion” or terms that confer a broader concept than gene conversion in their abstracts and/or keywords. For example, papers on “homologous recombination” or “reticulate evolution” can also deal with gene conversions and we will expand the search terms for future results.

Genetic research is increasing exponentially and our own trend analyses of gene conversion research (Figure 1) shows this. With this ever increasing amount of publications, the future of research will rely on data mining. In order to ease this process, it is important to include as much pertinent information in the abstracts as possible. This includes using exact species names, exact gene names, and exact disease and disorder names. This will greatly ease future research endeavors and allow for easier consolidation of research results.

As we tagged various terminologies related to gene conversion in all these abstracts, we have generated a large amount of data through this manual tagging process. We can use the data for both training and testing of our machine learning algorithms for predicting things such as the genes that are involved in gene conversion, the consequence of gene conversion at both gene sequence level and phenotypic level, that is, diseases or pathogenicity, using an abstract. Similarly, we can expand our work to mining the entire research paper. This way, we can create a database of gene conversion data including species, genes, and diseases/disorders.

Furthermore, we hope to use this data to facilitate in the identification of gene conversions. With the increasing amount of sequenced genomes, it would be ideal if we could use a software solution to automatically predict gene conversions. Our own research has used an ensemble of existing gene conversion identification programs in addition to rare-class learning techniques to identify gene conversions and the results have been promising [10]. However, more data on actual, proven gene conversions would greatly help with this. We can then expand gene conversion research across all types of species which in turn will lead to further understanding of this process.

## Figures and Tables

**Table 1:** Species Breakdown.

Species	Total Count	Mechanism	Event	N/A
Humans/*Homo sapiens *	642	115	522	6
Yeast/*Saccharomyces cerevisiae *	490	463	24	3
Mouse/*Mus musculus *	188	89	97	2
Chicken/*Gallus gallus *	89	54	35	0
Fruit Fly/*Drosophila melanogaster *	88	45	41	2
E. Coli/*Escherichia coli *	46	37	7	2
Rabbit/*Oryctolagus cuniculus *	46	22	23	1
Rat/*Rattus norvegicus *	34	4	30	0
*Trypanosoma brucei*	34	21	13	0
Fission yeast/*Schizosaccharomyces pombe *	31	26	4	1
Chimp/*Pan troglodytes *	20	2	17	1
Cow/*Bos taurus *	18	4	14	0
*Ascobolus immersus*	18	4	14	0
Chinese Hamster/*Cricetelus griseus *	16	16	0	0
Gonococci/*Neisseria gonorrhoeae *	16	11	4	1
*Arabidopsis thaliana*	15	8	7	0
Maize/*Zea mays *	15	6	9	0
*Plasmodium falciparum*	13	6	7	0
Salmonella/*Salmonella typhimirium *	11	6	5	0
Asexual Yeast/*Candida albicans *	11	7	4	0
Silk Moth/*Bombyx mori *	10	0	10	0
Tobacco/*Nicotania tabacum *	10	7	3	0
*Aspergillus nidulans*	9	7	2	0

In this table, we have sorted the found species based on the number of abstracts they were found in (listed here as Total Count). In addition we list the amount of abstracts that dealt with a mechanism of gene conversion, a specific gene conversion event, or whether we were unable to determine if it was either based on the information given (N/A).

**Table 2:** Superkingdom and Eukaryote Kingdom Breakdown.

Superkingdom	Species count	Paper count	Mechanism	Event	N/A
Eukaryotes	247	2030	990	1025	15
Bacteria	40	137	98	36	3
Viruses	15	30	22	8	0
Archaea	1	1	0	1	0

Kingdom	Species count	Paper count	Mechanism	Event	N/A

Metazoa	154	1268	372	887	9
Fungi	24	589	541	42	6
Viridiplantae	44	93	34	59	0

In this table, we list how many abstracts fall into superkingdom and kingdom categories (for the kingdom breakdown, we focus on eukaryotes). We list the total count of abstracts as well as a breakdown of how many of these abstracts dealt with gene conversion mechanisms, specific gene conversion events, or whether there was not enough information to determine the type of gene conversion research (N/A).

**Table 3:** Species Breakdown of Diversity and Conservation.

Species	ia	ia/div	ia/con	ig	ig/div	ig/con
Humans/*Homo sapiens *	152	142	1	149	22	69
Yeast/*Saccharomyces cerevisiae *	17	1	1	5	0	4
Mouse/*Mus musculus *	7	6	1	44	3	29
Chicken/*Gallus gallus *	1	1	0	19	4	12
Fruit Fly/*Drosophila melanogaster *	6	4	0	16	2	11
E. Coli/*Escherichia coli *	1	1	0	5	2	1
Rabbit/*Oryctolagus cuniculus *	1	1	0	7	2	4
Rat/*Rattus norvegicus *	0	0	0	19	1	15
*Trypanosoma brucei*	1	0	0	3	0	3
Fission yeast/*Schizosaccharomyces pombe *	2	0	0	0	0	0
Chimp/*Pan troglodytes *	3	2	1	11	2	5
Cow/*Bos taurus *	1	1	0	9	2	6
*Ascobolus immersus*	1	0	0	0	0	0
Chinese Hamster/*Cricetelus griseus *	0	0	0	0	0	0
Gonococci/*Neisseria gonorrhoeae *	0	0	0	0	0	0
*Arabidopsis thaliana*	0	0	0	5	0	5
Maize/*Zea mays *	0	0	0	5	1	4
*Plasmodium falciparum*	0	0	0	4	3	0
Salmonella/*Salmonella typhimirium *	0	0	0	4	0	3
Asexual Yeast/*Candida albicans *	1	1	0	0	0	0
Silk Moth/*Bombyx mori *	0	0	0	7	0	7
Tobacco/*Nicotania tabacum *	0	0	0	1	0	1
*Aspergillus nidulans*	0	0	0	0	0	0

In this table we list the species with the highest abstract counts and detail the type of gene conversion events they have undergone. Our focus here is on whether the conversion was between two distinct genes or two alleles from the same gene and whether the gene conversion event led to genetic diversity or gene conservation. Ia refers to an interallelic event and ia/div and ia/con refer to interallelic events that lead to genetic diversity and gene conservation, respectively. Ig refers to an intergenic event and ig/div and ig/con refer to intergenic events that lead to genetic diversity and gene conservation, respectively.

**Table 4:** Gene Conversion Diseases/Disorders Part 1.

Disease/Disorder	Gene 1	Gene 2	Pseudogene?	Papers
Phenylketunoria (PKU)	N/A	N/A	N/A	1
Huntington's Disease	N/A	N/A	N/A	3
Thalassemia	IVS-2	N/A	N/A	1
APRT Deficiency	APRT	N/A	N/A	1
Congenital Adrenal Hyperplasia/Hydroxylase Deficiency	CYP21	CYP21P	Yes (CYP21P)	77
Hereditary Persistence of Fetal Hemoglobin	protein S alpha	protein S beta	No	3
Debrisoquine polymorphism	CYPD6	CYPD7 (CYPD6∗2)	No	3
Sickle Cell Anemia	A Gamma	G Gamma	No	4
Gaucher's Disease	GBA	psGBA	Yes (psGBA)	6
Thrombocytopenia	HLA Class II	N/A	N/A	1
Haemoglobin H Disease	N/A	N/A	N/A	1
Rheumatalogic Disease	HLA complex	N/A	N/A	1
Beta Thallasemia	Beta-Globin Locus	N/A	N/A	3
Blue Cone Monochromacy	RCP	GCP	No	2
K36.16 thymoma	N/A	N/A	N/A	1
Rheumatoid Arthritis	DR4	N/A	N/A	1
Spinal Muscular Atrophy	SMN	SMNtel	No	16
Hypertension	CYP11B2	CYP11B1	No	3
Chronic Myeloid Leukaemia (CML)	ABL	N/A	N/A	1
Fragile X Syndrome	FMR1	FMRa/FRAXAC2	No	5
Homocysturnia	CBS	N/A	N/A	1
Von Willebrand Disease	VWF	N/A	Yes	5
Myotonic Dystrophy	N/A	N/A	N/A	3
Myeloma	GAU Hyprid Alpha	N/A	N/A	1
Human Complement C4A Deficiency	C4A	C4B	No	1
Neurofibriomatosis Type 1 (NF1)	NF1	NF1 pseudogene	Yes	3
Colorectal Cancer	APRT	N/A	N/A	2
Carbonic anhydrase II deficiency	CA II	N/A	N/A	1
Fanconic Anemia	FAC	N/A	N/A	2
Mucopolysaccharidosis type I Hurler/Scheie	alpha-L-iduronidase	N/A	N/A	1

In this table we list the diseases and disorders associated with gene conversions. In addition, we list the genes involved where applicable (listed here as Gene 1 and Gene 2) as well as whether one was a pseudogene (and listing which is if this information was available). Finally, we list the number of abstracts that dealt with the disease/disorder.

**Table 5:** Gene Conversion Diseases/Disorders Part 2.

Disease/Disorder	Gene 1	Gene 2	Pseudogene?	Papers
Hereditary Neuropathy with liability to Pressure Palsies	N/A	N/A	N/A	2
Charcot-Marie-Tooth disease type 1A	N/A	N/A	N/A	3
Polycistic Kidney Disease	PKD1	N/A	Yes	3
Autosomal dominant facioscapulohumeral muscular dystrophy	N/A	N/A	N/A	2
Breast Cancer	BRCA1	BRCA2	No	1
Hereditary Pancreatitis	PRSS1	R122H	No	5
non-Hodgkin's Lymphoma	D6S347	N/A	N/A	1
Spinocerebellar ataxia type 8	N/A	N/A	N/A	2
Neural Tube Defects	N/A	N/A	Yes	1
Friedreich's ataxia	N/A	N/A	N/A	1
Pseudoxanthoma elasticum	ABCC6	psiABCC6	Yes (psiABCC6)	1
Incontinentia pigmenti	NEMO/LAGE2	N/A	N/A	1
Schwachman Diamond Syndrome	SBDS	SBDSP	Yes (SBDSP)	6
Hypergonadotrophic Hypogonadism	FSHR	N/A	N/A	1
Smith-Magenis Syndrome	N/A	N/A	N/A	1
Human Male Infertility	DAZ genes	N/A	N/A	2
Hemophilia A	F8	N/A	N/A	1
Chronic Pancreatitis	PRSS1	PRSS2	No	1
Campomelic dysplasia	SOX9	N/A	N/A	1
Machado-Joseph Disease	MJD/SCA3	N/A	N/A	2
Sodium-sensitive cardiac hypertrophy	CYPB112	N/A	N/A	1
Obesity	HTR2C	N/A	N/A	1
Velo-cardio-facial syndrome/DiGeorge syndrome	LCR22-2	LCR22-4	No	1
Hereditary Nonpolyposis Colorectal Cancer	MLH1	MSH2	No	1
Atypical Hemolytic Uremic Syndrome	CFH	CFH1	No	1
Pyridoxine-responsive Homocystinuria	CBS	N/A	N/A	1
Autosomal Dominant Cataract	CRYBB2	CRYBB2P1	Yes (CRYBB2P1)	1

In this table we list the diseases and disorders associated with gene conversions. In addition, we list the genes involved where applicable (listed here as Gene 1 and Gene 2) as well as whether one was a pseudogene (and listing which is if this information was available). Finally, we list the number of abstracts that dealt with the disease/disorder.

**Table 6:** Further analyses.

Tagged data	Paper counts
Region	
Exon	12
Intron	7
5′-UTR	1
3′-UTR	0

Amount	
1-to-1	187
1-to-Many	3
Many-to-Many	36

Type	
Algorithm	4
Model	4

In this table we list additional data that was gathered in this project and the number of papers in each category. Region refers to the region of the gene on which the gene conversion occurred. Amount refers to the number of genes involved in gene conversion. Type refers to whether the papers dealt with algorithms or models.
